# Effects of vitamin D supplementation on a deep learning–based mammographic evaluation in SWOG S0812

**DOI:** 10.1093/jncics/pkae042

**Published:** 2024-05-30

**Authors:** Julia E McGuinness, Garnet L Anderson, Simukayi Mutasa, Dawn L Hershman, Mary Beth Terry, Parisa Tehranifar, Danika L Lew, Monica Yee, Eric A Brown, Sebastien S Kairouz, Nafisa Kuwajerwala, Therese B Bevers, John E Doster, Corrine Zarwan, Laura Kruper, Lori M Minasian, Leslie Ford, Banu Arun, Marian L Neuhouser, Gary E Goodman, Powel H Brown, Richard Ha, Katherine D Crew

**Affiliations:** Department of Medicine, Columbia University Irving Medical Center and the Herbert Irving Comprehensive Cancer Center, New York, NY, USA; Public Health Sciences Division, Fred Hutchinson Cancer Center, Seattle, WA, USA; SWOG Cancer Research Network, Statistics and Data Management Center, Seattle, WA, USA; Department of Radiology, Lenox Hill Hospital, New York, NY, USA; Department of Medicine, Columbia University Irving Medical Center and the Herbert Irving Comprehensive Cancer Center, New York, NY, USA; Department of Medicine, Columbia University Irving Medical Center and the Herbert Irving Comprehensive Cancer Center, New York, NY, USA; Department of Medicine, Columbia University Irving Medical Center and the Herbert Irving Comprehensive Cancer Center, New York, NY, USA; SWOG Cancer Research Network, Statistics and Data Management Center, Seattle, WA, USA; SWOG Cancer Research Network, Statistics and Data Management Center, Seattle, WA, USA; William Beaumont Hospital, Beaumont National Cancer Institute Community Oncology Research Program, Troy, MI, USA; Cancer Care Specialists of Central Illinois, Heartland National Cancer Institute Community Oncology Research Program, Decatur, IL, USA; William Beaumont Hospital, Beaumont National Cancer Institute Community Oncology Research Program, Troy, MI, USA; Department of Clinical Cancer Prevention, MD Anderson Cancer Center, Houston, TX, USA; Anderson Area Cancer Center, Southeast Clinical Oncology Research Consortium National Cancer Institute Community Oncology Research Program, Anderson, SC, USA; Lahey Hospital and Medical Center, Burlington, MA, USA; Department of Breast Oncology, City of Hope Medical Center, Duarte, CA, USA; Division of Cancer Prevention, National Cancer Institute, Bethesda, MD, USA; Division of Cancer Prevention, National Cancer Institute, Bethesda, MD, USA; Department of Clinical Cancer Prevention, MD Anderson Cancer Center, Houston, TX, USA; Public Health Sciences Division, Fred Hutchinson Cancer Center, Seattle, WA, USA; Swedish Cancer Institute, Pacific Cancer Research Consortium National Cancer Institute Community Oncology Research Program, Seattle, WA, USA; Department of Clinical Cancer Prevention, MD Anderson Cancer Center, Houston, TX, USA; Department of Medicine, Columbia University Irving Medical Center and the Herbert Irving Comprehensive Cancer Center, New York, NY, USA; Department of Medicine, Columbia University Irving Medical Center and the Herbert Irving Comprehensive Cancer Center, New York, NY, USA

## Abstract

Deep learning–based mammographic evaluations could noninvasively assess response to breast cancer chemoprevention. We evaluated change in a convolutional neural network–based breast cancer risk model applied to mammograms among women enrolled in SWOG S0812, which randomly assigned 208 premenopausal high-risk women to receive oral vitamin D3 20 000 IU weekly or placebo for 12 months. We applied the convolutional neural network model to mammograms collected at baseline (n = 109), 12 months (n = 97), and 24 months (n = 67) and compared changes in convolutional neural network-based risk score between treatment groups. Change in convolutional neural network-based risk score was not statistically significantly different between vitamin D and placebo groups at 12 months (0.005 vs 0.002, *P* = .875) or at 24 months (0.020 vs 0.001, *P* = .563). The findings are consistent with the primary analysis of S0812, which did not demonstrate statistically significant changes in mammographic density with vitamin D supplementation compared with placebo. There is an ongoing need to evaluate biomarkers of response to novel breast cancer chemopreventive agents.

Women at high risk for breast cancer are eligible for chemoprevention with selective estrogen receptor modulators or aromatase inhibitors, which have been shown in randomized controlled trials to reduce the incidence of invasive breast cancer by up to 50%-65% (1-6). However, chemoprevention uptake remains as low as 5% among high-risk women (7,8), and rates of early treatment discontinuation are as high as 40% (9), for reasons including treatment side effects (10,11).

Another potential barrier to the use of breast cancer chemoprevention is the lack of a short-term pharmacodynamic response biomarker that could demonstrate efficacy to patients (ie, reduction in breast cancer risk). With validation, a short-term biomarker of response might also serve as a surrogate for breast cancer incidence in chemoprevention trials, which could improve trial efficiency. Because high-risk women standardly receive annual mammography, mammography-based evaluations could serve as noninvasive biomarkers of response to chemoprevention. For example, mammographic density, or the proportion of radiodense glandular tissue on mammography, is a strong predictor of breast cancer risk (12,13). Short-term reduction in mammographic density might serve as a predictive biomarker of response to chemoprevention with tamoxifen (14). However, the use of mammographic density is limited by variability in radiologists’ visual interpretations (15), as well as lack of observed change in mammographic density among postmenopausal women who receive aromatase inhibitors (16-18).

Deep learning technologies applied to mammographic images might refine breast cancer risk prediction through evaluation of unique features beyond those visible to the human eye. Using a screening mammographic dataset of patients with known breast cancer (cases) and patients without breast cancer (controls), we developed a novel, fully automated convolutional neural network–based breast cancer risk model that evaluates unique mammographic features to provide a breast cancer risk score (19). The convolutional neural network model was a more accurate, independent predictor of breast cancer risk than mammographic density, with an overall accuracy of 72% in predicting breast cancer. Among a racially and ethnically diverse screening population, we observed that a hybrid model incorporating the convolutional neural network-based breast cancer risk model and the Breast Cancer Surveillance Consortium risk calculator more accurately predicted breast cancer than the Breast Cancer Surveillance Consortium model alone among Black and Hispanic women (20). We also applied the convolutional neural network model to serial mammograms among high-risk women and observed that women who received anti-estrogen chemoprevention compared with those who did not have a greater mean decrease in convolutional neural network score from baseline to 3-5 years of follow-up (21).

Further investigation requires application of the convolutional neural network-based breast cancer risk model to mammographic datasets among high-risk women, including those enrolled in clinical trials evaluating novel chemopreventive agents. In an exploratory analysis of a completed early phase chemoprevention trial of vitamin D, we hypothesized that vitamin D supplementation is associated with a greater decrease in convolutional neural network-based risk score compared with placebo among premenopausal women at high-risk for breast cancer. SWOG S0812 was a multicenter, randomized, double-blind, placebo-controlled trial among premenopausal women who met high-risk criteria for breast cancer, based on 5-year risk of invasive breast cancer of at least 1.67% and/or lifetime risk of at least 20%, history of atypical hyperplasia, lobular or ductal carcinoma in situ, prior stage 0–II breast cancer, a hereditary breast cancer syndrome, or high mammographic density (22). Participants had baseline serum 25-hydroxyvitamin D (25[OH]D) of no more than 32 ng/mL and were randomly assigned 1:1 to receive oral vitamin D3 20 000 international units (IU) weekly or placebo for 12 months. The primary endpoint was change in mammographic density at 12 months, measured using the Cumulus technique (23). Digital mammograms were collected at baseline, 12 months, and 24 months after random assignment, and serum biomarkers including vitamin D metabolites (25[OH]D), insulin-like growth factor 1 (IGF-1), and IGF binding protein3 were assessed at baseline, 6 months, and 12 months. This trial was approved by the SWOG central institutional review board, and informed consent was obtained for all participants.

We applied the convolutional neural network breast cancer risk model, as previously described (19,21), to bilateral mammograms obtained during S0812 to assess for absolute change in convolutional neural network score from baseline to 12 and 24 months. The output of the convolutional neural network model is expressed as a continuous variable (range = 0-1), with higher scores indicating higher predicted risk of breast cancer. This risk score does not incorporate clinical risk factors. We used 2-sample *t* tests to compare change in convolutional neural network score from baseline to 12 and 24 months among women who received vitamin D supplementation vs placebo. We calculated Pearson correlation coefficients to assess for correlation between change in convolutional neural network score at 12 months and change in mammographic density and serum biomarkers at 12 months. We conducted multivariable linear regression analyses to assess factors associated with 1) baseline convolutional neural network-based breast cancer risk score and 2) change in convolutional neural network-based risk score. We also assessed the relationship by treatment arm and change in convolutional neural network-based risk score, adjusting for age, body mass index (BMI), race and ethnicity, mammographic density, and serum biomarkers. The level of statistical significance was a *P* value less than .05 for all analyses. Analyses were conducted using SAS OnDemand for Academics (Cary, NC, USA).

Baseline characteristics of the 208 enrolled participants were previously reported (22). Median age was 44.6 years (range = 21-50 years), and 77.4% of participants identified as non-Hispanic White, 6% as Black, and 8% as Hispanic. Median BMI was 25.9 kg/m^2^ (range = 18.6-46.5 kg/m^2^). Baseline characteristics were similar between treatment arms.

Baseline convolutional neural network scores, mammographic density, and serum biomarkers, and change in these measures at follow-up, are shown in Table 1. Among the 208 enrolled women, 109 had evaluable baseline mammograms for analysis using the convolutional neural network model of whom 97 had evaluable mammograms at 12 months and 67 had evaluable mammograms at 24 months. Mean baseline mammographic density was similar between vitamin D and placebo groups (38.1% vs 35.4%, *P* = .332) as were mean baseline convolutional neural network scores (0.219 vs 0.190, *P* = .234). There was no statistically significant difference between groups in change in convolutional neural network risk score from baseline to 12 months (0.005 vs 0.002, *P* = .875) or in change in convolutional neural network risk score from baseline to 24 months (0.020 vs 0.001, *P* = .563). As previously reported (22), the differences in change in mammographic density from baseline to follow-up (12 or 24 months) between groups were not statistically significant. Receipt of vitamin D supplementation was not statistically significantly associated with change in convolutional neural network risk score at 12 months after adjusting for age, BMI, race and ethnicity, and change in mammographic density at 12 months.

**Table 1.** Convolutional neural network-based breast cancer risk scores, mammographic density, and serum biomarkers at baseline and change from baseline to follow-up among women enrolled in SWOG S0812, stratified by treatment arm

Outcome	Timeframe	Vitamin D		Placebo		*P* ^a^
		No.	Mean (SD)	No.	Mean (SD)	
Convolutional neural network-based risk score	Baseline	57	0.219 (0.167)	52	0.190 (0.113)	.234
	Change at 12 months	50	0.005 (0.092)	47	0.002 (0.120)	.875
	Change at 24 months	30	0.020 (0.143)	37	0.001 (0.114)	.563
Mammographic density, %	Baseline	84	38.12 (17.16)	73	35.44 (17.26)	.332
	Change at 12 months	81	−0.55 (7.66)	73	−0.12 (7.95)	.732
	Change at 24 months	67	−1.39 (8.22)	58	0.96 (10.38)	.160
Serum 25-hydroxyvitamin D, ng/mL	Baseline	96	25.43 (10.73)	91	24.19 (8.59)	.382
	Change at 6 months	85	18.03 (17.85)	77	4.30 (9.11)	**<.001**
	Change at 12 months	62	18.66 (18.32)	57	3.38 (9.91)	**<.001**
Serum insulin-like growth factor 1, ng/mL	Baseline	96	15.89 (30.69)	92	17.14 (31.66)	.783
	Change at 6 months	85	−6.47 (41.80)	78	6.72 (34.29)	**.029**
	Change at 12 months	63	−10.86 (46.21)	57	−0.37 (34.65)	.166
Serum insulin-like growth factor binding protein 3, µg/mL	Baseline	96	5.10 (1.02)	92	5.04 (0.96)	.640
	Change at 6 months	85	0.06 (0.68)	78	0.11 (0.64)	.660
	Change at 12 months	63	−0.20 (0.74)	57	0.07 (0.81)	.056

a Using 2-sample *t* tests. Bolded value indicates *P* value <.05, considered statistically significant.

We also evaluated if baseline convolutional neural network risk score and change in convolutional neural network risk score at 12 months were associated with age, BMI, mammographic density, and serum biomarkers. There was a statistically significant positive correlation between baseline convolutional neural network-based risk score and age (correlation coefficient *R* = 0.288, *P* = .0024) but no significant correlation between baseline convolutional neural network-based risk score and BMI or baseline mammographic density. There was no significant correlation between baseline convolutional neural network-based risk score and baseline serum 25(OH)D, IGF-1, or IGF binding protein 3. In a multivariable linear regression model adjusting for BMI, baseline mammographic density, and baseline serum 25(OH)D (Table 2), a 1-year increase in age was associated with a 0.007 increase in baseline convolutional neural network score (*P* = .002). However, there was no statistically significant association between change in convolutional neural network-based risk score and age, BMI, change in mammographic density, or change in serum 25(OH)D at 12 months in univariable or multivariable analyses, adjusting for baseline convolutional neural network-based risk score.

**Table 2.** Multivariable linear regression analyses evaluating associations between baseline convolutional neural network-based breast cancer risk score and change in convolutional neural network risk score at 12 months and other variables, including age, mammographic density, and serum 25-hydroxyvitamin D [25(OH)D]^a^

Variable	Baseline convolutional neural network-based risk score		Change in convolutional neural network-based risk score at 12 months	
	Estimate (95% CI)	*P*	Estimate (95% CI)	*P*
Age, y	0.007 (0.003 to 0.012)	**.002**	−0.002 (−0.006 to 0.434)	.056
Body mass index, kg/m^2^	0.004 (−0.001 to 0.008)	.132	−0.002 (−0.005 to 0.002)	.410
Baseline convolutional neural network-based risk score			−0.450 (−0.618 to -0.282)	**<.001**
Baseline mammographic density	0.001 (0.001 to 0.003)	.108		
Baseline serum 25(OH)D, ng/mL	−0.002 (−0.005 to 0.001)	.246		
Change in mammographic density at 12 mo, %			−0.001 (−0.004 to 0.001)	.257
Change in serum 25(OH)D, at 12 mo, ng/mL			−0.001 (−0.003 to 0.002)	.470

a CI = confidence interval. Bolded value indicates *P* value <.05, considered statistically significant.

In summary, we applied the convolutional neural network–based breast cancer risk model to prospectively obtain mammograms from high-risk premenopausal women enrolled in SWOG S0812 and found that change in convolutional neural network-based breast cancer risk score at 12 months was not statistically significantly different between women who received vitamin D supplementation compared with placebo. Our findings are consistent with the primary results of S0812, which did not find a statistically significant difference in short-term change in mammographic density with vitamin D supplementation compared with placebo (22).

Although the lack of observed change in the convolutional neural network-based risk score could support the primary conclusion of S0812 that there is insufficient evidence for the use of vitamin D supplementation for breast cancer risk reduction, it remains unknown whether nonhormonal interventions result in change in mammography-based assessments, including convolutional neural network-based risk score and mammographic density and whether these assessments should be used as surrogates for breast cancer risk to assess novel chemopreventive agents. Given that a barrier to the development of novel breast cancer chemoprevention strategies is the large sample sizes and years of follow-up required to report outcomes such as breast cancer incidence, future studies utilizing prospectively obtained mammograms from chemoprevention trials are necessary to evaluate potential short-term biomarkers of response to nonhormonal chemopreventive agents.

We did not observe any statistically significant association between change in convolutional neural network score at 12 months and change in serum biomarkers, including IGF-1, IGF binding protein3, and vitamin D metabolites. Baseline convolutional neural network-based risk score had a statistically significant positive correlation with patient age, consistent with the known increase in breast cancer risk with increasing age (24). We did not find statistically significant associations between baseline convolutional neural network-based risk score and baseline mammographic density or between changes in convolutional neural network-based risk score and mammographic density, possibly because the convolutional neural network model evaluates mammographic features beyond quantification of mammographic density . Further investigation of potential associations between convolutional neural network-based risk score and imaging- and/or blood-based biomarkers of breast cancer risk could provide potential insight into convolutional neural network–based risk prediction but will require larger patient cohorts.

Our analysis also highlights the need for improved systems to collect breast imaging for translational substudies within prospective multicenter clinical trials. Only approximately half of participants in S0812 had evaluable mammograms for analysis using the convolutional neural network-based model at baseline and 12 months. Notably, only approximately two-thirds of participants had evaluable mammograms for analysis of change in mammographic density at 12 months, the primary endpoint of S0812. Although we do not have patient-level information on reasons for the greater proportion of unevaluable mammograms using the convolutional neural network-based model compared with mammographic density, inadequate image quality for analysis using the convolutional neural network-based model and issues with central upload and transfer of images to the analyzing center might have contributed. Efficient methods for collection of breast imaging among participants in clinical trials will be essential to the evaluation of potential imaging-based biomarkers of response to chemoprevention.

Additional limitations of our analysis that could limit generalizability include use of digital mammograms but not digital breast tomosynthesis, which is increasingly used in the clinical setting. We do not have information on mammography vendors for available images. Also, participants in S0812 were predominantly non-Hispanic White women, which could limit generalizability to diverse populations. Overall, the small sample size of participants with evaluable mammograms limited the statistical power of our analyses and might have contributed to the lack of observed associations between change in convolutional neural network-based risk score and vitamin D supplementation, as well as change in serum biomarkers and mammographic density.

In conclusion, vitamin D supplementation did not result in a statistically significantly different change in convolutional neural network-based breast cancer risk score compared with placebo among high-risk premenopausal women enrolled in S0812. Further evaluation of the convolutional neural network model as a potential biomarker of response to breast cancer chemoprevention will require use of large, prospectively obtained mammographic datasets among high-risk women who receive selective estrogen receptor modulators or aromatase inhibitors, as well as nonhormonal chemopreventive agents. There is still an unmet clinical need to evaluate potential short-term biomarkers of response to nonhormonal chemopreventive agents, which could be used to accelerate development of novel prevention strategies.

## Data Availability

The data generated in this study are available upon request from the corresponding author.
